# Confronting Zoonoses, Linking Human and Veterinary Medicine

**DOI:** 10.3201/eid1204.050956

**Published:** 2006-04

**Authors:** Laura H. Kahn

**Affiliations:** * Princeton University, Princeton, New Jersey, USA

**Keywords:** Zoonosis, veterinarians, physicians, perspective

## Abstract

Many of the emerging infectious diseases, including those caused by bioterrorist agents, are zoonoses. Since zoonoses can infect both animals and humans, the medical and veterinary communities should work closely together in clinical, public health, and research settings. In the clinical setting, input from both professions would improve assessments of the risk-benefit ratios of pet ownership, particularly for pet owners who are immunocompromised. In public health, human and animal disease surveillance systems are important in tracking and controlling zoonoses such as avian influenza virus, West Nile virus, and foodborne pathogens. Comparative medicine is the study of disease processes across species, including humans. Physician and veterinarian comparative medicine research teams should be promoted and encouraged to study zoonotic agent-host interactions. These efforts would increase our understanding of how zoonoses expand their host range and would, ultimately, improve prevention and control strategies.

## Abstract 2

Greater collaboration is needed between human and veterinary medicine to better control zoonoses.

Zoonoses are diseases that can be transmitted from wild and domestic animals to humans and are public health threats worldwide. Because these diseases come from animals, prevention and control strategies need to be innovative and require the combined efforts of many fields. For example, closer collaborations are needed between veterinarians, physicians, and public health professionals in 3 areas: individual health, population health, and comparative medicine research. In the individual health setting, assessing the potential for zoonotic disease transmission from animals to humans should include input from both physicians and veterinarians, especially for patients at high risk such as those who are immunocompromised. In population health, zoonotic disease threats should be addressed through surveillance systems that include domestic and wild animal and human populations, which would help lead to effective control measures. Since physicians and veterinarians would be the key professionals to recognize and report outbreaks, enhanced communications between hospital epidemiologists, veterinarians, and local public health officials would not only help expedite a local response, but also help identify whether unusual diseases or outbreaks involving animals and humans were related or separate events. In the research setting, collaboration between physicians and veterinarians in comparative medicine would improve our understanding of zoonotic agent-host interactions.

## Individual Health Collaborations

At the individual health level, zoonotic diseases are a concern for all who live or work with animals. This risk is especially problematic for persons, such as companion animal owners, who are immunocompromised. Grant and Olsen found that physicians are generally not comfortable discussing the role of animals in the transmission of zoonoses and would prefer that veterinarians play a role (1). However, most patients do not view veterinarians as a source of information for human health. The authors found that only 21% of HIV patients asked their veterinarians about the health risks of pet ownership (1).

Zoonotic risks from companion animals are not limited to those living with HIV. One patient who was taking infliximab for longstanding rheumatoid arthritis became infected with *Cryptococcus neoformans* after cleaning a cockatiel's cage the week before hospitalization (2). Human lymphocytic choriomeningitis virus (LCMV) infection is associated with pet rodents and also causes serious infections in immunocompromised persons (3). These risks extend beyond the pet owners and can involve the recipients of the animal owners' donated organs. For example, LCMV has been responsible for the deaths of 3 organ transplant recipients who received their organs from donors who had owned infected pet rodents (3).

Exotic or unusual pets can pose a risk to the healthy. Salmonellosis developed in 4 children, 1 mother, and an 80-year-old woman after exposure to small pet turtles (4). Salmonellosis has also been associated with pet rodents. For example, during the summer of 2004, two young children became seriously ill with salmonellosis shortly after their families purchased pet rodents (5). A national search of the PulseNet National *Salmonella* Database from December 2003 to October 2004 found 28 matching human-case isolates of *Salmonella enterica* serovar Typhimurium. Of the 22 patients who were interviewed, 13 (59%) had been exposed to rodents during the 8 days before onset of illness (5).

Exotic pets can introduce pathogens previously unknown in the North American continent. For example, the 2003 monkeypox outbreak in the Midwestern United States originated after imported African rodents infected prairie dogs in pet distribution facilities (6). Laboratory-confirmed monkeypox developed in 35 persons (6). No one died, but the outbreak required vaccinating 30 persons with smallpox vaccine, 23 because of potential occupational exposure (6).

Occupational risks for exposure to zoonotic diseases are a concern for persons such as farmers, meatpackers, and pet shop employees who work with animals. For example, *Streptococcus suis* can cause meningitis or occasionally fulminant sepsis in pig farmers (7,8) *Campylobacter* infection is an occupational risk for packers in poultry factories, and *Streptobacillus moniliformis* can be an occupational risk for pet shop employees (9,10)

These examples illustrate that living and working with animals can impact human health at the individual level. Veterinarians who treat animals that suddenly become ill with confirmed infections should assess the risk for zoonotic potential and inform the animals' owners accordingly. From a medical-legal standpoint, veterinarians are obligated to do this, but the extent to which they should inform animal owners and ensure that they seek medical attention varies depending on the circumstances (11). The severity of the risk for zoonotic disease as well as the level of understanding by the animal owner in question would need to be considered (11). For example, the veterinarian may merely advise potentially exposed persons to seek medical attention or may strenuously urge and ensure that the person receives medical attention immediately. However, veterinarians' roles in assessing risk for potential zoonotic disease transmission could extend beyond this level of involvement.

Risk-benefit ratios for ongoing animal exposure could be weighed and discussed by both veterinarians and physicians. The roles in these veterinary-physician relationships would need to be established from the start so that the veterinarians would not be at risk of appearing to practice medicine. For example, veterinarians could provide an assessment of an animal's health status to a physician whose patient is immunocompromised and insists on keeping his or her companion animal. Since companion animal ownership has psychologic and physiologic benefits, this type of collaboration and cooperation between the 2 professions would be invaluable to patients. The veterinarian would provide regular checkups to the companion animal to ensure that its health status is closely monitored. In the occupational setting, regular veterinary monitoring of all involved animals' health may not be possible; however, if a worker were immunocompromised, then a careful assessment should be made about his or her continuing that line of work. Veterinary input might be helpful in these difficult decisions. Joint medical and veterinary medical workshops on zoonotic risks to human health could help forge ties and facilitate opportunities to establish these types of collaborative efforts.

## Population Health Collaborations

Recognizing whether human and animal outbreaks were simultaneous would provide important information for identifying the causative pathogens and developing control strategies. For example, physicians treating the initial West Nile virus (WNV) patients in New York City in 1999 might have benefited if they knew that for the previous month and concurrently, veterinarians in the surrounding area had been seeing dozens of dying crows with neurologic symptoms similar to those of the affected humans (12). Depending on the state, animal disease surveillance can be fragmented. For example, in New York, human and animal rabies are the responsibility of local and state health departments, livestock are overseen by the state agriculture agency, and wildlife is the responsibility of the state environmental agency (12).

In New York, no local or state agency assumed full responsibility for the large wildlife die-off investigation in 1999 since which agency was responsible was not initially clear (12). This situation hindered communications between the veterinarians, public health officials, and physicians who were involved in the outbreak response at the local level. As an emergency, short-term measure, veterinarians could have expressed their concerns directly to the hospital epidemiologists in the area to be on the lookout for a possible human impact from an unknown disease that was causing widespread severe neurologic symptoms and death in wild birds. Such rapid, direct communication between veterinarians and physician epidemiologists could be particularly important in states in which local public health agencies either do not exist or are not involved in zoonotic disease reporting or investigation.

In some states, animal disease reporting and response are state level functions and are separate from human public health. I contacted state veterinarians in all 50 states about their states' animal disease reporting requirements. State veterinarians from 8 (19%) of the 43 responding states replied that veterinarians are required to contact their local public health agencies directly about reportable zoonotic diseases. Of these, 2 require reports of rabies only. Names and contact information were obtained from the US Department of Agriculture Animal and Plant Health Inspection Service website (http://www.aphis.usda.gov/vs/sregs/official.html) and the Council of State and Territorial Epidemiologists Point of Contact Veterinarians website (http://www.cste.org/). State agencies such as departments of agriculture, environment, or boards of animal health are the usual primary recipients of animal disease reports. However, these agencies may not have the resources to conduct animal disease prevention and control activities at the local level. In addition, in the case of departments of agriculture, their mission, historically, has been to promote agriculture, not necessarily to control infectious diseases in all types of animals.

Animal disease reporting and oversight are split between different agencies in some states. This is the situation at the federal level and has prompted a recent National Academy of Sciences report to recommend that a federal-level, centralized coordinating mechanism be established to improve collaboration and cooperation among all the players in animal health oversight, including industry and local, state, and federal agencies (13). A similar mechanism for improving communication and collaboration across state agencies, such as between state animal health and public health veterinarians, would be important since evidence suggests that veterinarians preferentially report to more "animal-centric" state agencies.

For example, the Alaska Department of Health and Social Services and the Alaska Department of Environmental Conservation (DEC) mailed a laboratory usage and needs assessment survey to all 200 licensed veterinarians in Alaska. Of the 140 who responded, 95% stated that they would report to the state veterinarian at DEC, 4% to the state department of health, and 1% to the US Department of Agriculture when asked, "Who would you contact if you suspected or diagnosed a reportable animal disease?" (R. Gerlach, pers. comm.).

In 2004, I surveyed 4,144 randomly selected licensed veterinarians in 4 states: New Hampshire, New Jersey, New York, and Pennsylvania. When asked, "Which government agency would you first notify if your companion animal or livestock patient had an unusual infectious disease?" the largest percentage of the 1,070 respondents chose "State Agriculture Agency." Some veterinarians, ≈10% for companion animals and 14% for livestock, would skip the state and local agencies altogether and notify a federal agency. Twenty-eight percent of the veterinarians did not know if their community had a local public health agency. The survey did not include questions about wildlife. Veterinarians' names and addresses were obtained from each state's licensing boards except for New York State, which prohibits access to this information. For New York State, names and addresses were obtained from the American Veterinary Medical Association (Tables 1 and 2).

**Table 1:** Veterinarians' choices of government agencies they would first notify regarding a companion animal patient with an unusual infectious disease*

Choice	No. (%)
State agriculture agency	326 (30)
State public health agency	241 (23)
Local public health agency	206 (19)
Other†	116 (11)
Federal agency (USDA, FDA, CDC)	111 (10)
Not sure	30 (3)
Do not care for companion animals	27 (3)
Did not answer question	13 (1)
Total	1,070 (100)

*USDA, US Department of Agriculture; FDA, Food and Drug Administration; CDC, Centers for Disease Control and Prevention. †A total of 76 (66%) of 116 said, "state veterinarian," 20 (17%) of 116 gave a combination of government agencies, and 20 (17%) of 116 gave miscellaneous answers, including Animal Plant Health Inspection Service, state veterinary diagnostic laboratory, and animal hospital employer.

**Table 2:** Veterinarians' choices of government agencies they would first notify regarding a livestock animal with an unusual infectious disease*

Choice	No. (%)
State agriculture agency	422 (40)
US Department of Agriculture	141 (13)
Do not take care of livestock	135 (13)
State public health agency	122 (11)
Other†	118 (11)
Local public health agency	57 (5)
Not sure	42 (4)
Did not answer question	17 (2)
Other federal agency (CDC, FDA, FBI)	16 (1)
Total	1,070 (100)

*CDC, Centers for Disease Control and Prevention; FDA, Food and Drug Administration, FBI, Federal Bureau of Investigation. †A total of 77 (65%) of 118 said, "state veterinarian," 25 (21%) of 118 gave a combination of government agencies, and 16 (14%) of 118 said that they do not see livestock.

In addition to working with state officials during serious zoonotic outbreaks, veterinarians should also communicate and collaborate with local public health officials. During the 1999 WNV outbreak, the presumptive diagnoses for the initial human cases included Guillain-Barré syndrome, encephalitis, meningitis, and aspiration pneumonia (14). Public health officials assumed the cause of the outbreak was St. Louis encephalitis (SLE) until a veterinary pathologist at the Bronx Zoo linked the animal and human outbreaks (12). She realized that crows and other birds ordinarily resistant to SLE were dying, so the agent was not likely SLE. Her work helped set the stage for the discovery of WNV in the Western Hemisphere (12).

At the population level, zoonotic pathogens cause foodborne, waterborne, and arthropodborne disease outbreaks. These pathogens include *Salmonella*, *Escherichia coli* O157:H7, *Cryptosporidium*, yellow fever virus, and *Borrelia burgdorferi* (15). Many of the category A, B, and C bioterrorist agents, such as *Bacillus anthracis*, *Yersinia pestis*, *Francisella tularensis*, *Coxiella burnetii*, and Nipah virus, cause zoonoses (16,17).

The magnitude of the problem of zoonoses illustrates why the efforts of medicine, veterinary medicine, and public health need to overlap. Taylor and others identified 1,415 infectious agents and found that 868 (61%) could be transmitted between animals and humans (18). They found that zoonotic diseases were twice as likely to be associated with emerging or newly discovered infections than nonzoonotic pathogens and that viruses and protozoa were the zoonotic pathogens most likely to emerge. RNA viruses, in particular, have been identified as highly likely to emerge (19). These agents include WNV, avian influenza virus, hantavirus, and severe acute respiratory syndrome–associated coronavirus.

Joint surveillance of animal and human zoonotic disease outbreaks is already reaping benefits worldwide. For example, recognition of the first human case of H5N1 avian influenza in Hong Kong in 1997 was facilitated by the surveillance of ducks, geese, and chickens in southern China during the preceding decades (20). On the domestic front, in 1999, the Centers for Disease Control and Prevention established ArboNET, a cooperative surveillance system that monitors the geographic spread of WNV in humans, mosquitoes, birds, and other animals in response to the outbreak of WNV disease (21). ArboNET has provided an invaluable system for tracking the disease's spread and severity across the United States, identifying early WNV activity, and justifying continuing support for mosquito control (22). These types of surveillance systems should be continued and expanded to include other serious zoonotic diseases such as plague and tularemia.

In addition to ongoing joint surveillance activities, researchers should collaborate in applied public health studies. For example, physician and veterinarian teams could conduct serosurveys of humans who live and work near high-risk animal populations to assess their risk of acquiring zoonoses. Long-term surveillance studies could be conducted on humans who are exposed to deer and elk, which are at risk of acquiring chronic wasting disease in disease-endemic regions of Colorado, Wyoming, and Nebraska (23). Surveillance studies on the role of vaccinated and unvaccinated horses in the amplification of WNV to humans would help improve our understanding of the epidemiology of virus activity (24).

## Comparative Medicine Research Collaborations

The need for physicians and veterinarians to work together to control zoonoses extends beyond the individual and population health settings and should include collaborations in comparative medicine research. Comparative medicine is the study of the anatomic, physiologic, and pathophysiologic processes across species, including humans. Considerable attention is paid to infectious diseases, specifically the study of host-agent interactions.

As an academic discipline, comparative medicine is not new; the first chair in it was established in 1862 in France (25). The field has an illustrious history. In 1893, Theobald Smith, a physician, and F.L. Kilbourne, a veterinarian, published a paper establishing that an infectious agent, *Babesia bigemina*, the cause of cattle fever, was transmitted by an arthropod vector (25). Their seminal work helped set the stage for Walter Reed's discovery of yellow fever transmission (25). Another physician-veterinarian team, Drs. Rolf Zinkernagel and Peter C. Doherty, won the 1996 Nobel Prize in physiology or medicine for their discovery of how the immune system distinguishes normal cells from virus-infected cells (26).

These 2 examples illustrate that medicine and veterinary medicine are complementary; they are synergistic in generating new scientific insights across species. In essence, the 2 disciplines epitomize the philosophy of comparative medicine. And yet, as societies' needs grow to have scientists work together to understand and control emerging zoonoses, evidence suggests that the next generation of medical and veterinary medical scientists are not collaborating with each other. Biomedical and comparative medicine research is losing its appeal as a career among physicians and veterinarians.

On the physician side, the decline in physician-scientists is evidenced by several trends. First, from 1970 to 1997, the number of physician-scientists obtaining National Institutes of Health (NIH) support has been essentially flat and shrinking in proportion to doctoral recipients who seek and obtain funding (27). Second, from 1994 to 1997, the number of first-time physician-scientists seeking NIH funds dropped by 31%, and the percentage of medical school graduates interested in research careers fell from 14% in 1989 to 10% in 1996 (27). Medical school faculties now comprise 25% fewer physician-scientists than 20 years ago (28).

For veterinarian-scientists, the situation is considered dire. A 2004 National Academy of Sciences (NAS) report found that of American Veterinary Medical Association members, <1% were board certified in laboratory animal medicine and <2% were board certified in pathology (29). In addition, the total number of veterinarians who receive NIH grant funding is small. In 2001, only 4.7% of all NIH grants funded for animal research were awarded to veterinarian principal investigators (29).

Reasons for the lack of interest in research are similar for both medical and veterinary students: an emphasis on clinical care, educational debt, and a lack of mentors and research opportunities (30,31). Medical schools now emphasize primary care and care for the underserved, and while certainly important, this shift in priorities has been at the expense of encouraging biomedical research careers.

Veterinary schools have shifted their focus from comparative medicine research and livestock medicine to companion animal medicine to meet societal demand (32). However, similar to the situation with medical schools, this shift has caused fewer numbers of veterinary students to pursue research careers. In addition, comparative medicine programs have been shifting from a research to service orientation that limits veterinarians' research involvement to being primarily caretakers for laboratory animals (32). This shift in comparative medicine orientation has discouraged many veterinary students from pursuing careers in research and hinders research on emerging zoonoses from diverse animal hosts.

What can be done? Although NIH has begun a roadmap to improve biomedical research into the 21st century, nowhere does the plan mention comparative medicine and the importance of veterinary involvement, which would certainly fit into its goals of promoting interdisciplinary research and new pathways to discovery (33). An NAS report recognizes the need for the roadmap initiative to address this issue and recommends creating integrated veterinary research through joint interagency collaborative programs at NIH (34).

One way to achieve this would be to offer jointly sponsored comparative medicine research grants from both the National Center for Research Resources (NCRR) and National Institute of Allergy and Infectious Diseases (NIAID). With NIAID's emphasis on zoonoses and cross-species investigations, comparative medicine research would fit in well with its mission of research on bioterrorist agents, emerging infectious diseases, and immunology. The NCRR and NIAID could offer research grants to medical and veterinary medical research teams that are promoting collaborative projects on zoonoses.

A second NAS report addressing this issue recommends that federal agencies involved in human and animal research coordinate their efforts. Jointly funded integrated and comprehensive animal health research programs should be established to ensure that veterinary and medical scientists work together as collaborators domestically and internationally (13).

Encouraging more veterinary school graduates to pursue careers in research is critical if partnerships are to be developed. A third NAS report recommends that the number of veterinarians serving as principal investigators should increase (29). This could be accomplished by increasing the number of NCRR-funded T32 training grants and making them available to persons who want to enter research training programs immediately after graduation from veterinary school (29). Finally, another way to encourage more veterinary students to pursue research careers would be for the National Institute of General Medical Sciences (NIGMS) to offer research training programs to them analogous to those offered to medical students. Currently, NIGMS research training programs are only open to holders of MD and PhD degrees (35).

## Discussion

Since zoonoses are diseases of animals that can infect humans, veterinarians, physicians, and public health officials need to work more closely together to control, prevent, and understand them. In the individual health setting, collaborative input from both veterinarians and physicians would help assess a patient's potential zoonotic disease risks from animal exposure. For high-risk immunocompromised patients, these collaborative efforts could be tremendously important, not only for their personal well-being but also for their livelihoods.

Regarding population health, reporting of animal diseases varies considerably from state to state. Some states have 1 agency responsible for all animal disease reporting while others split the reports between various agencies. However, in many states, animal disease surveillance appears to be largely a state level function. In few states, local public health agencies are expected to receive zoonotic disease reports directly from veterinarians. If controlling zoonotic diseases is to be improved, greater communication and collaboration between veterinarians, physicians, and public health officials at the local level are needed. One NAS report recommends a federal level mechanism to promote greater collaboration among all the players involved in animal health (13); similar mechanisms could also be considered in states.

Joint disease surveillance efforts, which are proving to be extremely useful in the tracking of zoonoses, include ArboNet for WNV surveillance, the National Antimicrobial Resistance Monitoring System for enteric bacteria surveillance, and FoodNet for the population-based surveillance of foodborne pathogens (36,37). These programs should continue to be supported, and new surveillance programs for other serious zoonoses should be developed. Medical, veterinary, and public health schools should offer courses on zoonotic risks to human health that integrate all 3 perspectives.

Society would benefit if more collaborative comparative medicine research projects were conducted by physicians and veterinarians to investigate zoonotic agent-host interactions. Among the many ways to promote these projects are multiagency-sponsored comparative medicine research grants and more training grants for veterinarians interested in careers in research. These efforts would increase our understanding of how zoonoses expand their host range and would, ultimately, improve prevention and control strategies.
